# Low-Temperature Fabrication of Stable Black-Phase CsPbI_3_ Perovskite Flexible Photodetectors Toward Wearable Health Monitoring

**DOI:** 10.1007/s40820-024-01565-4

**Published:** 2024-11-15

**Authors:** Yingjie Zhao, Yicheng Sun, Chaoxin Pei, Xing Yin, Xinyi Li, Yi Hao, Mengru Zhang, Meng Yuan, Jinglin Zhou, Yu Chen, Yanlin Song

**Affiliations:** 1 College of Chemistry and Pingyuan Laboratory, Zhengzhou University, Zhengzhou, 450001 People’s Republic of China; 2 Key Laboratory of Green Printing, Institute of Chemistry, Chinese Academy of Sciences, Beijing, 100190 People’s Republic of China; 3 Key Laboratory of Bio-Inspired Materials and Interfacial Science, Technical Institute of Physics and Chemistry, Chinese Academy of Sciences, Beijing, 100190 People’s Republic of China; 4 Institute of High Energy Physics, Chinese Academy of Sciences, Beijing, 100049 People’s Republic of China

**Keywords:** In situ hydrolyzation, Low-temperature processing, All-inorganic perovskite, Flexible photodetectors, Health monitoring

## Abstract

Flexible wearable optoelectronic devices fabricated from organic–inorganic hybrid perovskites significantly accelerate the development of portable energy, biomedicine, and sensing fields, but their poor thermal stability hinders further applications. Conversely, all-inorganic perovskites possess excellent thermal stability, but black-phase all-inorganic perovskite film usually requires high-temperature annealing steps, which increases energy consumption and is not conducive to the fabrication of flexible wearable devices. In this work, an unprecedented low-temperature fabrication of stable black-phase CsPbI_3_ perovskite films is demonstrated by the in situ hydrolysis reaction of diphenylphosphinic chloride additive. The released diphenyl phosphate and chloride ions during the hydrolysis reaction significantly lower the phase transition temperature and effectively passivate the defects in the perovskite films, yielding high-performance photodetectors with a responsivity of 42.1 A W^−1^ and a detectivity of 1.3 × 10^14^ Jones. Furthermore, high-fidelity image and photoplethysmography sensors are demonstrated based on the fabricated flexible wearable photodetectors. This work provides a new perspective for the low-temperature fabrication of large-area all-inorganic perovskite flexible optoelectronic devices.

**Supplementary Information:**

The online version contains supplementary material available at 10.1007/s40820-024-01565-4.

## Highlights

- Low-temperature fabrication of black-phase CsPbI_3_ perovskite films is first demonstrated by using diphenylphosphinic chloride additive under 30–50 °C, arising from the steric effect and chloride insertion engineering.
- Large-area high-quality all-inorganic perovskite films with fewer defects enhanced crystallographic orientation, and excellent environmental stability is fabricated.
- The record performances are demonstrated for flexible wearable photodetectors with a responsivity of 42.1 A W^−1^, a detectivity of 1.3 × 10^14^ Jones, high-fidelity image, photoplethysmography sensor functions, and high mechanical stability.

**Supplementary Information:**

The online version contains supplementary material available at 10.1007/s40820-024-01565-4.

## Graphical abstract

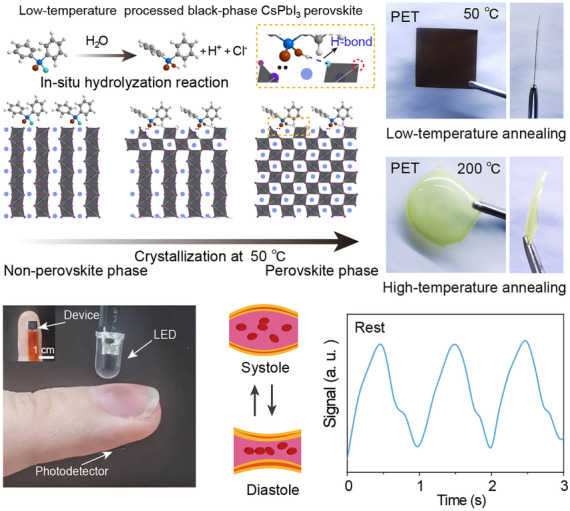

## Introduction

Flexible wearable optoelectronic devices with lightweight and bendable features display considerable application prospects in the fields of biomedicine, sensing/imaging, robotics, health monitoring, etc. [1–8]. In particular, metal halide perovskite materials have emerged as a powerful candidate for flexible wearable devices owing to their high optoelectronic performance, low-cost solution processing, and excellent compatibility with flexible substrates [9–17]. Nowadays, flexible wearable devices made of organic–inorganic hybrid perovskites (OIHPs) have achieved remarkable advancement, but their poor thermal stability arising from the volatility of the organic components within OIHPs impedes commercialization [17–23]. In contrast, all-inorganic perovskite materials CsPbI_3_ with good chemical components stability and suitable bandgap are more tolerant to temperature, but they usually present a non-perovskite hexagonal polymorph (*δ*-phase) with a bandgap of about 2.82 eV at room temperature due to their unideal tolerance factor [24–29]. Furthermore, the massive defects within the all-inorganic perovskite films impede the performance of optoelectronic devices [28, 30]. Thus, the fabrication of stable black-phase all-inorganic CsPbI_3_ perovskite with fewer defects is a critical prerequisite for achieving high-performance optoelectronic devices.

To achieve the fabrication of stable black-phase all-inorganic CsPbI_3_ perovskite, a series of solution chemistry approaches were demonstrated by the introduction of polymers [31, 32], anion alloying [33], surface functionalization [34–36], etc. For example, stable black-phase CsPbI_3_ perovskite films were successfully fabricated by introducing polymers, including polyvinyl pyrrolidone (PVP) [37] and poly(ethylene oxide) (PEO) [31], which can be attributed to the effective passivation of defects and the decreased crystal domain size by the addition of polymers. For anion alloying, by substituting iodide ions (I^−^) with chloride ion (Cl^−^) and bromide ions (Br^−^), stable black-phase perovskite films are obtained, but the alloying of the ions results in an increased band gap and reduced photovoltaic performance [33]. The introduction of large organic ammoniums, such as phenethylammonium iodide (PEAI) [34], 2-(naphthalene-1-yl)ethanamine (NEA) [35], and ethylenediamine (EDA) [38], also enables the fabrication of black-phase perovskite films by surface functionalization. However, the fabrication processes of black-phase CsPbI_3_ perovskite films often require a high-temperature annealing step, which increases the fabrication cost, energy consumption, and the processing difficulty of flexible wearable devices and tandem solar cells. To achieve low-temperature processing, the vacuum thermal evaporation method was developed, but it still requires complex equipment and stringent growth conditions, thus limiting its large-area device application [30, 39]. Therefore, the low-temperature solution growth of all-inorganic black-phase CsPbI_3_ perovskite films toward flexible wearable devices is still challenging.

In this work, we first achieve flexible wearable devices based on the low-temperature-processed black-phase *γ*-CsPbI_3_ perovskite films by the one-step spin coating technology under 30–50 °C. The low-temperature phase transition process is mainly attributed to the in situ hydrolysis reaction of diphenylphosphinic chloride (DPPOCl) additive with water, which releases chloride ions and diphenyl phosphate (DPPOH), reducing the crystallization energy barrier of black-phase CsPbI_3_ perovskite. The generated chloride ions and DPPOH not only suppress the generation of non-perovskite phases but also efficiently passivate the halogen defects on the surface of perovskite films. Based on high-quality perovskite films, high-performance photodetectors were successfully fabricated with a responsivity of 42.1 A W^−1^ and a detectivity of 1.3 × 10^14^ Jones. Furthermore, flexible wearable photodetectors with good mechanical stability were realized based on low-temperature solution processing, which achieved high-fidelity imaging and PPG sensors. This work opens up a new perspective for high-performance flexible optoelectronic devices based on all-inorganic CsPbI_3_ perovskite films, which will facilitate large-scale commercialization of flexible optoelectronic devices.

## Experimental Section

### Materials

*N*, *N*-dimethylformamide (DMF, ≥ 99.9%), lead(II) iodide (PbI_2_, 99.999%), and PET substrate were purchased from Advanced Election Technology Co. Ltd. Cesium iodide (CsI, 99.999%) was purchased from Xi'an Yuri Solar Co., Ltd. Dimethyl sulfoxide, anhydrous (DMSO, ≥ 99.9%), cesium carbonate (Cs_2_CO_3_, 99.9%), valeric acid (C_5_H_10_O_2_, ≥ 99%), poly(methyl methacrylate) ((C_5_H_8_O_2_)_n_) were purchased from Sigma-Aldrich company. Diphenylphosphinic chloride (DPPOCl, 97 + %) was purchased from Alfa company. Ethanol (C_2_H_6_O), acetone (C_3_H_6_O), and 2-propanol (C_3_H_8_O) were purchased from Innochem company. All chemicals were used without further purification.

### Fabrication of All-Inorganic Perovskite Films

The all-inorganic perovskite precursor solution (0.6 M) was prepared by dissolving equal molar masses of CsI and PbI_2_ into a mixed solution of DMSO and DMF. For the perovskite film without the DPPOCl additive, the perovskite films were fabricated by spin coating at 3000 rpm for 45 s and high-temperature annealing under 350 °C for 5 min. For the perovskite film with DPPOCl additive (0.25% volume ratio relative to perovskite solution), perovskite films were deposited by spin coating at 3000 rpm for 45 s and low-temperature annealing under 30–50 °C for 5 min. To further enhance the crystalline quality of the film, 5% molar cesium valerate was added to the precursor solution.

### Characterization

The crystallinity of perovskite films with different fabrication conditions was measured by an X-ray diffractometer (XRD, Bruker, D8 focus, Germany). The morphology of perovskite films was collected by scanning electron microscope (SEM, Hitachi, S-8010, Japan) provided by eceshi (www.eceshi.com). Absorption spectra were obtained by UV–vis-NIR spectrometer (Cary 7000, Agilent, America). Photoluminescence (PL) spectra and lifetimes were acquired by Edinburgh Instruments (FLS1000, England). The binding energy of the elements was obtained by X-ray photoelectron spectroscopy (XPS) Instrument (ThermoFisher Scientific, ESCALABXi + , England). Fourier transform infrared (FTIR) spectral curves were acquired by FTIR spectroscopy (Thermo Scientific, Nicolet iS10, America). The grazing-incidence wide-angle X-ray scattering (GIWAXS) data were measured at 1W1A Diffuse X-ray Scattering Station, Beijing Synchrotron Radiation Facility (BSRF-1W1A).

### Device Fabrication and Performance Measurement

Before the fabrication of the photodetectors, the SiO_2_/Si substrate was cleaned sequentially with ethanol, acetone, and isopropanol. Then, the perovskite films were spin coated onto the surface of the SiO_2_/Si or PET substrate. The photoconductive devices consist of two metal electrodes (10 nm Cr, 100 nm Au) with channel lengths of 50 μm and width of 10 μm (distance between metal electrodes), respectively. PMMA was further spin coated onto the surface of perovskite films. The *I-V* curves of the devices were measured using a vacuum manual probe station (Lake Shore) and a 4200 semiconductor characterization system (Keithley, 4200). The light source is a 680-nm light-emitting diode (LED), and the intensity is calibrated by a silicon photodiode (S130C, Thorlabs). Switching stability and response speed were measured by a digital oscilloscope (DPO 4104, Tektronix). Noise currents were measured with a current preamplifier (SR570, Stanford Research Systems) and a lock-in amplifier (SR860, Stanford Research Systems).

## Results and Discussion

### Design of Flexible Wearable PPG Sensor Based on Low-Temperature-Processed All-Inorganic Perovskite Films

**Fig. 1:**
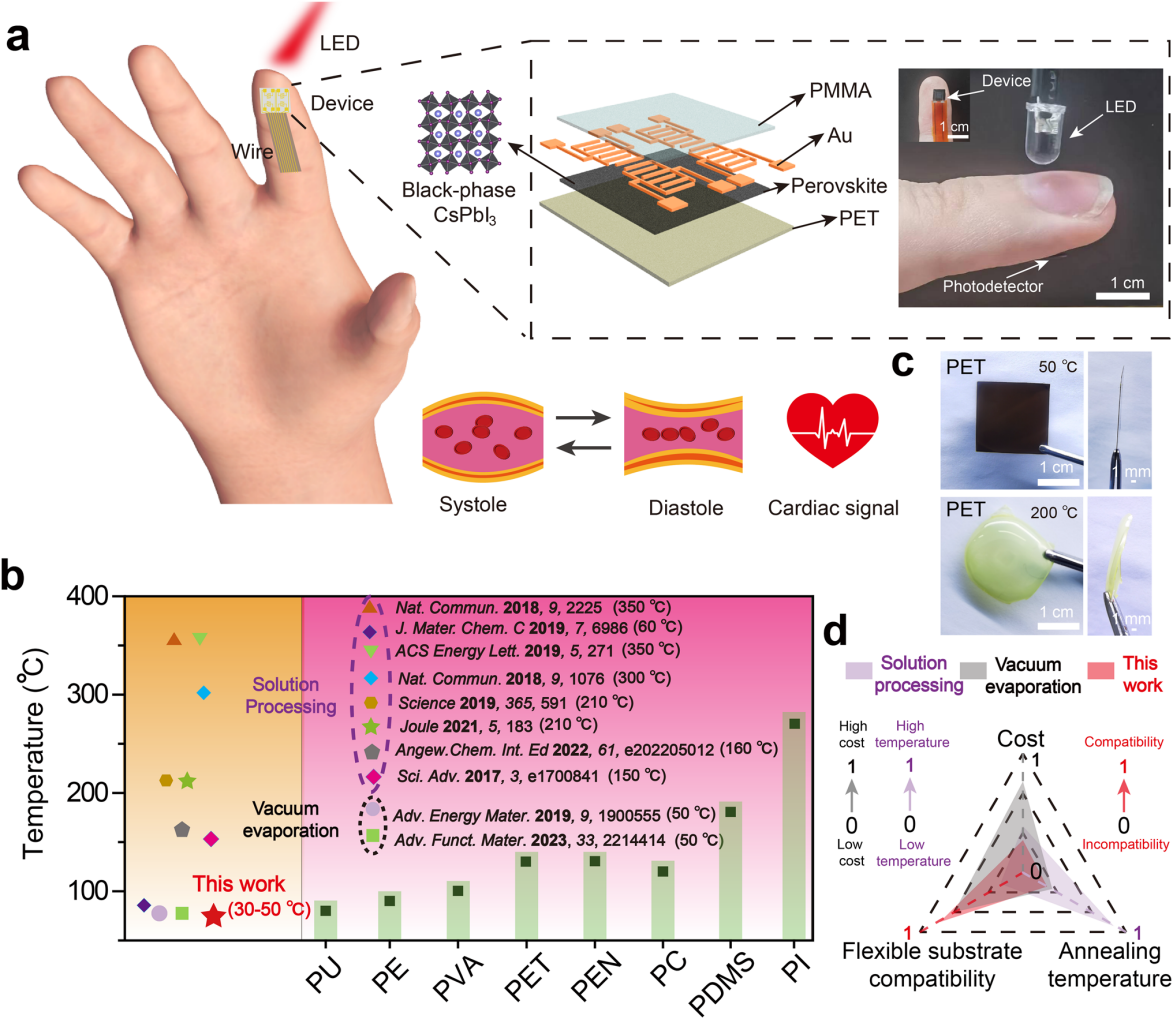
Schematic of flexible wearable PPG sensor based on low-temperature-processed all-inorganic perovskite films.** a** Schematic diagram of the working mechanism of the PPG sensor in transmissive mode. The dashed box shows a structure schematic of the PPG device including PET substrate, perovskite active layer, Au electrode, and PMMA barrier layer (left) and a real photograph of the blood pulse test (right).** b** Statistics of deformation temperatures of typical flexible substrates (pink-shaded regions) and phase transition temperatures of black-phase perovskites (yellow-shaded regions). **c** Photographs of flexible PET substrates under low-temperature annealing for perovskite film with DPPOCl additive (top) and high-temperature annealing for perovskite film without DPPOCl additive (bottom). **d** Comparison of advantages and disadvantages of different processing methods

PPG sensing is a non-invasive health monitoring technology, which is commonly used for monitoring cardiovascular health status, such as heart rate, blood pressure, and oxygen saturation [10]. According to the position of the light source and the sensor, PPG sensing can generally be classified as transmissive and reflective modes. Figure 1a shows a schematic and actual photograph of the PPG sensor in transmissive mode, including the flexible wearable all-inorganic perovskite photodetector, the red LED, and the finger. The flexible wearable photodetector consists of a flexible PET substrate, an all-inorganic perovskite film, gold electrodes, and a polymethyl methacrylate (PMMA) blocking layer. PMMA layer can not only prevent moisture and oxygen from damaging the perovskite film but also avoid the leakage of lead ions to protect the human body. When the red light passes through the finger to reach the flexible photodetector, the transmission intensity of the red light changes periodically with the systole and diastole of the blood vessels, thus enabling the detection of the human pulse signal for disease analysis [10]. Typically, the blood vessels dilate when the heart contracts, resulting in a weakening of the transmitted light. In contrast, the blood vessels contract when the heart diastoles, resulting in a strengthening of the transmitted light.

To comfortably and tightly adhere devices to the fingers or wrist, flexible devices need to be fabricated. However, the processing of all-inorganic perovskite devices usually requires a high-temperature annealing process, which is not conducive to the fabrication of flexible film devices due to the deformation temperature of most transparent flexible substrates being below 150 °C. Polyurethane, polyethylene, polyvinyl alcohol, polyethylene glycol terephthalate, polyethylene naphthalate two formic acid glycol ester, polycarbonate, polydimethylsiloxane, and polyimide are abbreviated as PU, PE, PVA, PET, PEN, PC, PDMS, and PI, respectively. Therefore, the fabrication of high-quality black-phase all-inorganic perovskite films using a low-temperature solution method needs to be addressed. Figure 1b provides a chart showing the temperature tolerance of typical flexible substrates and the phase transition temperature based on different fabrication methods for the black-phase all-inorganic perovskite. Although the vapor-phase method enables the low-temperature fabrication of all-inorganic perovskite films, its large equipment and complicated growth technology are not conducive to the fabrication of large-area flexible optoelectronic devices. To achieve the low-temperature fabrication of black-phase all-inorganic perovskite films, a new processing route was developed by adding DPPOCl into the precursor solution of perovskite. Figure 1c shows low-temperature and high-temperature annealed all-inorganic perovskite flexible films based on PET substrates. For the perovskite film with the DPPOCl additive, the low-temperature annealed films show a typical black phase, and no deformation for the PET substrate is revealed. On the contrary, the perovskite films without DPPOCl additive present a yellow non-perovskite phase with severe deformation, which proves the importance of low-temperature processing of all-inorganic perovskite films for the fabrication of flexible optoelectronic devices. Overall, compared to traditional solution methods and vacuum thermal evaporation methods, lower annealing temperatures, lower processing costs, and better flexibility compatibility have been demonstrated in our method (Fig. 1d).

### Mechanism and Characterization of Low-Temperature-Processed All-Inorganic Perovskite Films

**Fig. 2:**
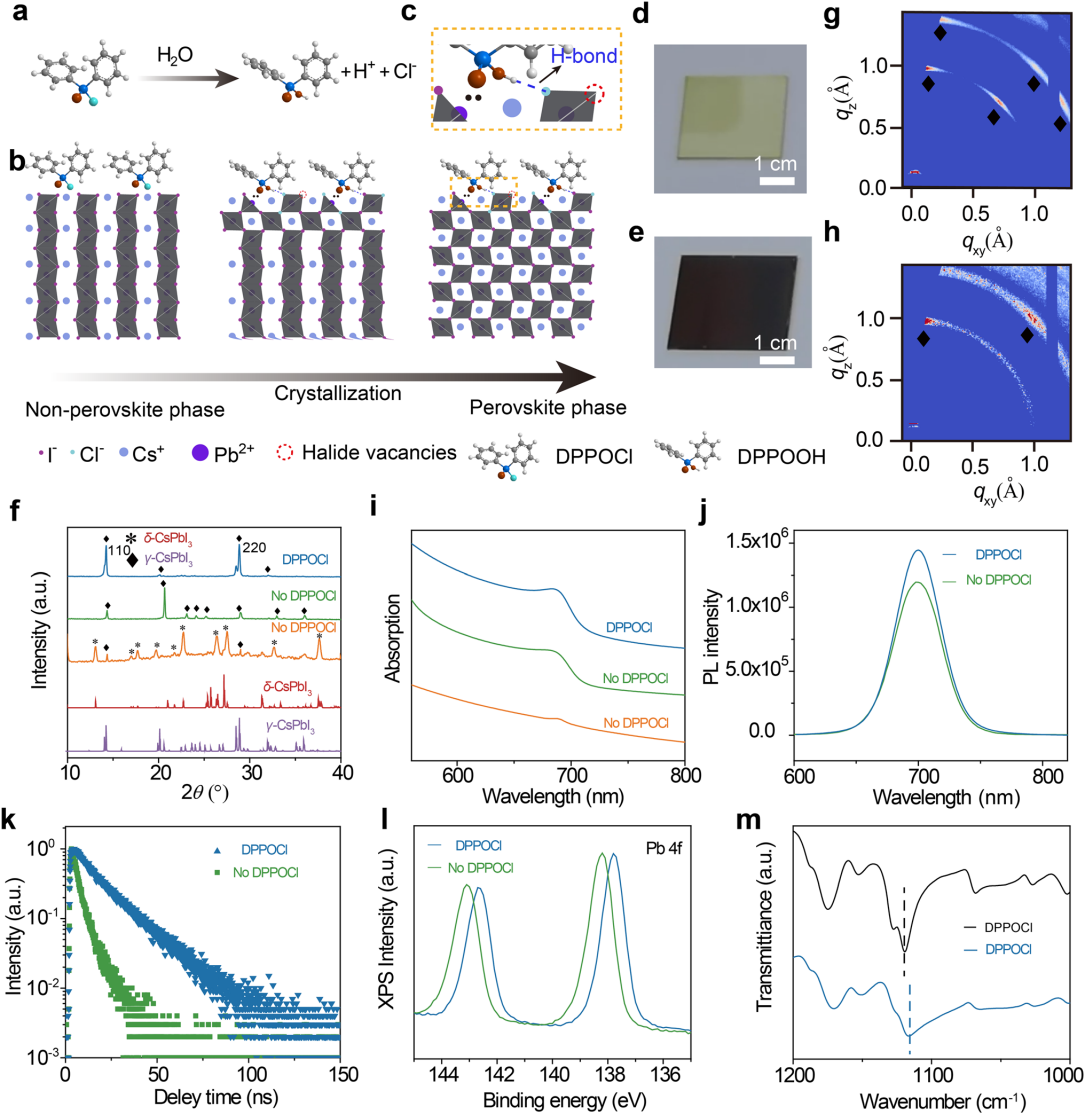
Mechanism, crystallinity, and spectral characterization of low-temperature phase transition from non-perovskite *δ*-phase to *γ*-CsPbI_3_ phase. **a** The release process of chloride ions and DPPOH based on the hydrolysis reaction of DPPOCl and water molecules. **b** Schematic diagram of the phase transition from yellow-phase to black-phase perovskite film, in which the introduction of chloride ions and DPPOH significantly reduces the crystallization energy barrier of black-phase perovskite and passivates the defects within the perovskite film. **c** Partial enlargement of the **b** image shows P = O: Pb coordination interaction and O–H–I hydrogen bonds. Photographs of the DPPOCl-treated films change from **d** yellow-phase to **e** black-phase perovskite. **f** XRD, **g-h** Giwaxs patterns of perovskite films with DPPOCl and without DPPOCl additive. **i** Absorption spectra under different fabrication conditions. The green line and yellow line represent perovskite films with DPPOCl additive annealed at 350 and 50 °C, respectively. **j** PL and **k** time-resolved PL spectroscopy of perovskite films with DPPOCl and without DPPOCl additive, indicating that the introduction of DPPOCl can significantly passivate the defects within the film. **l** XPS spectra perovskite films with DPPOCl and without DPPOCl additive. The significant shifts of Pb 4f peaks indicate strong coordination between P = O bonds and Pb^2+^. **m** The FTIR spectra result of P = O bonds for perovskite film with DPPOCl additive (blue line) and DPPOCl film (black line)

Although black-phase all-inorganic CsPbI_3_ perovskite films have remarkable temperature stability, their solution process usually requires a high-temperature annealing process, which not only significantly increases energy consumption, but also is not conducive to the fabrication of large-area flexible perovskite devices. With the introduction of DPPOCl, we achieved low-temperature solution fabrication of black-phase all-inorganic *γ*-CsPbI_3_ perovskite films for the first time. The introduction of DPPOCl presents the following advantages. First, a minor amount of DPPOCl will affect the crystallization and nucleation of perovskite films, yielding dense, pinhole-free films with small grain sizes, which can stabilize the black-phase perovskite [39]. Second, the steric effect of DPPOH released by the reaction of DPPOCl with trace moisture can significantly reduce the crystallization energy barrier of black-phase perovskite, thus leading to the low phase transition temperature [28, 38]. Third, the DPPOH and chloride ions can significantly reduce the surface defects and deep energy level defects of perovskite and enhance the device performance [40–43]. Fourth, compared to iodide ions, chloride ions have stronger bonding energy with lead ions, which can significantly inhibit the transformation of the black phase to the non-perovskite phase [33]. Figures 2a and S1 show the experimental process of low-temperature fabrication of black-phase all-inorganic CsPbI_3_ perovskite films and the chemical reaction processes of the hydrolysis reaction. The process of low-temperature phase transition is shown in Fig. 2b. In the condition of trace moisture and low-temperature annealing, the released chloride ions/DPPOH are first inserted/adsorbed to the octahedral frameworks of the non-perovskite phase, inducing the transition from a *δ*-phase to black-phase perovskite, which can be attributed to the decreased crystallization energy barrier due to the steric hindrance changing the connection of octahedral frameworks and the doping of chloride ions further improves the tolerance factor of black-phase perovskite [28, 33, 44, 45]. In addition, the released chloride ions and DPPOH enable excellent passivation of halogen defects on the surface and grain boundaries of perovskite films due to the formation of P = O: Pb bonds (Fig. 2c) [40, 46]. The color and crystal structure differences before and after the phase transformation of all-inorganic CsPbI_3_ perovskite films are shown in Figs. 2d, e, and S2, S3, which are consistent with the previously reported literature.

To investigate the crystallinity and the spectral properties of low-temperature-processed all-inorganic CsPbI_3_ perovskite films, XRD, GIWAXS, absorption spectra, photoluminescence spectra (PL), and PL lifetime were measured. Figure 2f shows the XRD patterns of perovskite films under different processing conditions. The XRD peaks without DPPOCl additive are mainly attributed to the non-perovskite *δ*-CsPbI_3_ phase with only a small amount of black-phase CsPbI_3_ existing within the film under low-temperature processing conditions. At high temperatures, the sample without DPPOCl additive can be completely converted to black-phase CsPbI_3_ films, but its (110) and (220) diffraction peaks are relatively weak and the films are poorly oriented. In contrast, the low-temperature-processed films with DPPOCl additive showed a perfectly *γ*-CsPbI_3_ diffraction peak with obvious (110) crystallographic orientation and enhanced crystallinity, indicating that the addition of DPPOCl enables the low-temperature fabrication of high-quality black-phase all-inorganic *γ*-CsPbI_3_ perovskite films (Figs. 2f and S4). Figure S5 further demonstrates that the fabricated perovskite films belong to the *γ*-CsPbI_3_ phase. Giwaxs patterns further confirm the enhanced crystallinity and crystallographic orientation for the low-temperature-processed films with DPPOCl additive (Fig. 2g, h). The weak absorption edge at 718 nm and the strong absorption below 450 nm further demonstrate that the complete black-phase conversion cannot be achieved in the low-temperature-processed films without DPPOCl additive, whereas only black-phase *γ*-CsPbI_3_ perovskite absorption peak is found for perovskite films with DPPOCl additive (Figs. 2i and S6) [24, 30, 47]. Therefore, the fully converted black-phase *γ*-CsPbI_3_ perovskite films can be attributed to the introduction of the DPPOCl additive. Furthermore, compared to the high-temperature-processed black-phase perovskite films, the low-temperature-processed perovskite films exhibit higher PL intensity and longer PL lifetime, indicating enhanced crystallinity and fewer defects arising from effectively defects passivation by DPPOH and chloride ions generated through hydrolysis reaction (Fig. 2j, k). To explain the defect passivation effect of DPPOH, XPS and FTIR were measured. The addition of DPPOCl results in a significant peak shift for the binding energy of the Pb_4f_ and I_3d_, indicating coordination interactions between the P = O bonds and Pb^2+^, thus passivating the halogen vacancies in the perovskite films (Figs. 2l and S7). This interaction is further confirmed by the peak shift of the P = O bonds in the FTIR result (Fig. 2m) [41, 42]. Hydrogen-bonding interaction between − OH functional groups and halide ions was also demonstrated (Fig. S8). In general, the introduction of DPPOCl not only realizes the low-temperature processing of black-phase *γ*-CsPbI_3_ perovskite films but also effectively passivates the defects within the perovskite films, which can contribute to the fabrication of high-performance flexible optoelectronic devices.

**Fig. 3:**
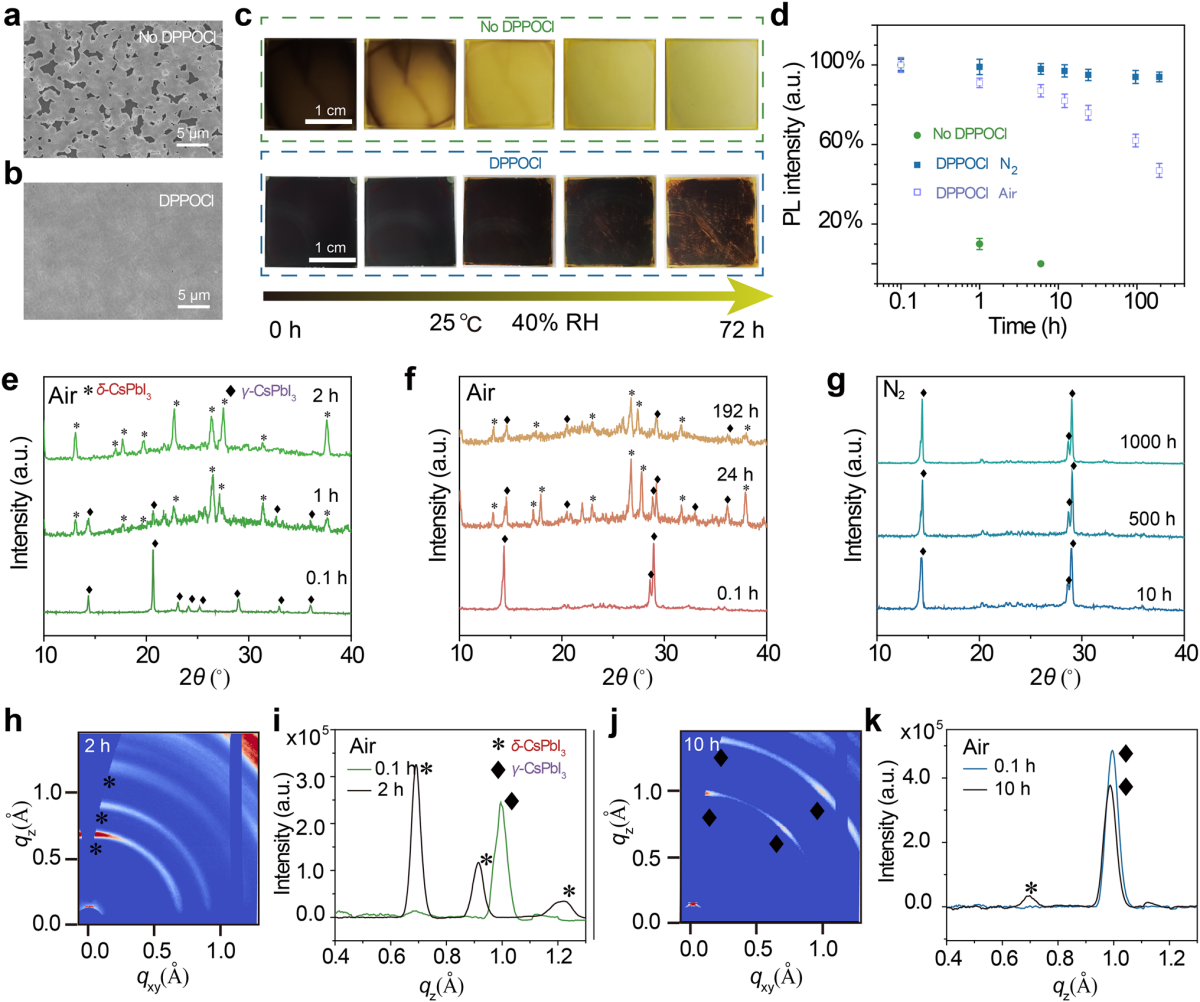
Stability characterization of perovskite films with DPPOCl and without DPPOCl additive. SEM images of perovskite films **a** without DPPOCl and **b** with DPPOCl additive.** c** Photographs of perovskite films with DPPOCl and without DPPOCl additive stored in ambient conditions with a relative humidity of 40% at 25 °C for different times. **d** The evolution of PL intensity of perovskite films with DPPOCl and without DPPOCl additive after storing different times at nitrogen, and air atmosphere. Error bars represent standard deviation. The evolution of XRD intensity of perovskite films **e** without DPPOCl, **f** with DPPOCl additive under air atmosphere and **g** with DPPOCl additive under nitrogen atmosphere. Giwaxs results of perovskite films **h, i** without DPPOCl, and **j, k** with DPPOCl additive under air atmosphere, demonstrating the excellent phase stability for perovskite films with DPPOCl additive (humidity: 40%; temperature: 25 °C)

The environmental stability of the all-inorganic *γ*-CsPbI_3_ perovskite films is an important factor for the performance of optoelectronic devices, so we monitored the environmental stability of all-inorganic *γ*-CsPbI_3_ perovskite films. The morphology of perovskite films is closely related to their environmental stability; thus, we measured the morphology of the films. The morphology of the perovskite films under different experimental conditions is shown in Figs. 3a, b, and S9. The morphology of perovskite film without DPPOCl additive presents numerous pinholes that can be attributed to the rapid crystallization process under high-temperature annealing. Due to the massive defects at the pinhole, a rapid phase transformation occurs under the invasion of moisture. On the contrary, the perovskite films with DPPOCl additive prepared at low temperatures exhibit a dense morphology, pinhole-free and smaller grain size, which is conducive to the stability of the black-phase *γ*-CsPbI_3_ perovskite films [34, 48]. To compare the environmental stability of the perovskite films, we systematically monitored the changes of the perovskite films at different times under a relative humidity of 40% and a temperature of 25 °C. The sample without the DPPOCl additive rapidly turned into a yellow non-perovskite phase, while the sample with DPPOCl exhibited excellent environmental stability without significant degradation after 72 h (Fig. 3c). PL intensity measurements further showed that the film with DPPOCl additive had excellent environmental stability, maintaining more than 50% of PL intensity stored in air for 192 h, while the PL intensity without DPPOCl disappeared after one hour. In addition, the samples with the DPPOCl additive maintained more than 95% PL intensity after 60 days in an inert atmosphere, indicating its strong intrinsic structural stability (Figs. 3d and S10). The XRD and Giwaxs patterns further demonstrate the enhanced phase stability for perovskite films with DPPOCl additive; only a slight decrease in peak intensity and a small amount of *δ*-phase was observed after tens of hours under air (Fig. 3e-k). The introduction of DPPOCl not only lowers the phase transition temperature but also greatly stabilizes the structure of black-phase *γ*-CsPbI_3_ perovskite due to its small grain size and effective defect passivation that prevents the erosion of moisture.

### Characterization of the Detection Performance

**Fig. 4:**
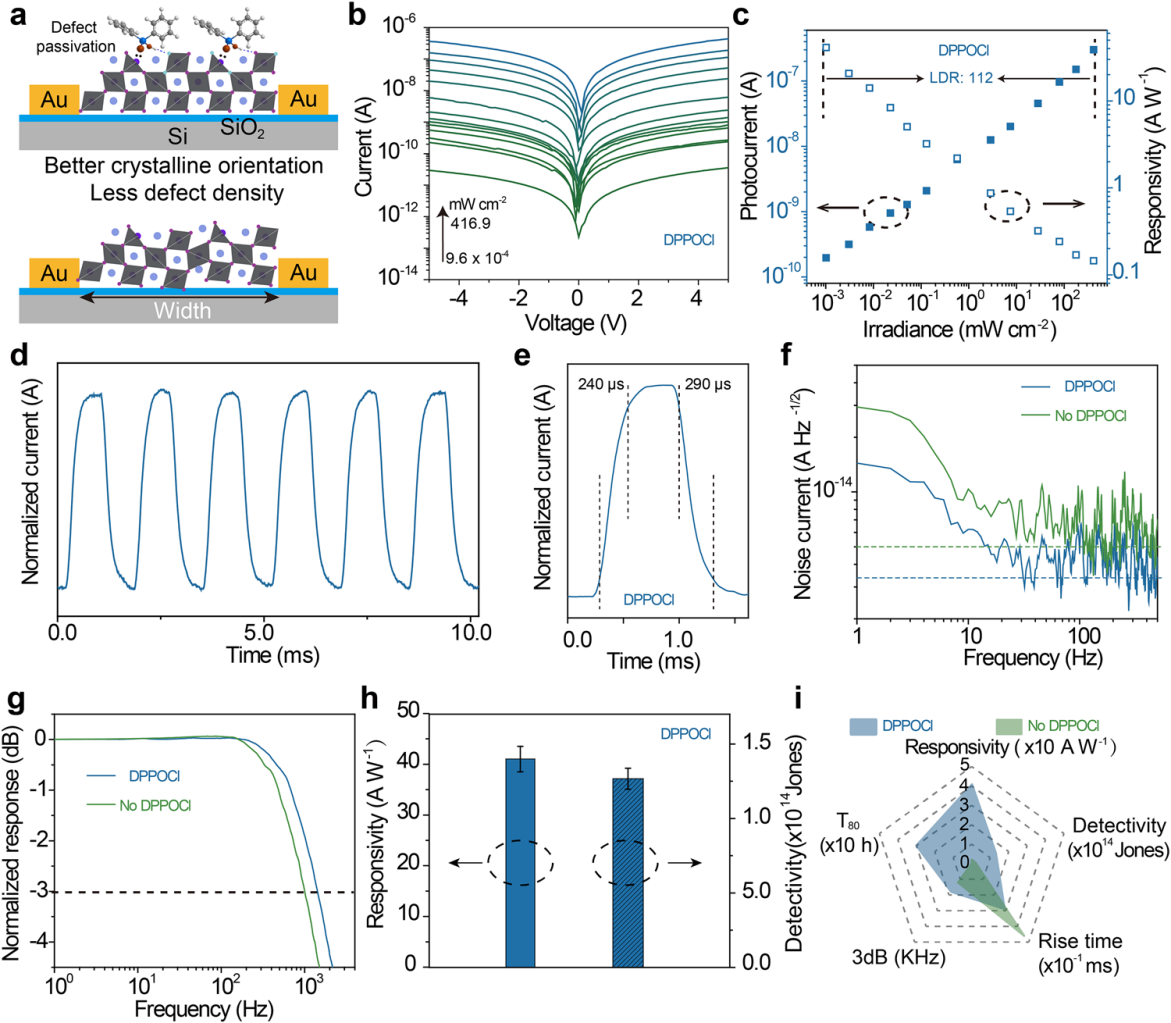
Photodetection performance of low-temperature-processed perovskite films. **a** Schematic diagram of a photoconductive photodetector. Low-temperature-processed devices (top), high-temperature-processed devices (bottom). **b** Logarithmic *I-V* curve of the photodetector based on perovskite devices with DPPOCl additive. **c** Photocurrents and responsivities of perovskite devices with DPPOCl additive under different irradiation powers ranging from 9.6 × 10^–4^ to 416.9 mW cm^−2^. **d** On–off stability of perovskite devices with DPPOCl additive under a fixed irradiation power of 416.9 mW cm^−2^. **e** Response speed under a fixed irradiation power of 416.9 mW cm^−2^. **f** The noise current and **g** normalized response curves of perovskite devices with DPPOCl and without DPPOCl additive under different frequencies. **h** Statistical responsivities and detectivities from ten different devices under a fixed light intensity of 9.6 × 10^–4^ mW cm^−2^. Error bars represent standard deviation. **i** Performance comparison of perovskite devices with DPPOCl and without DPPOCl additive

Based on the high-quality black-phase *γ*-CsPbI_3_ perovskite films with fewer defects, we fabricated two-terminal photoconductive photodetectors with Au as the electrode. The schematic diagram of the photodetector is shown in Fig. 4a. Figure 4b shows the log *I-V* curves of the photodetector under different irradiation powers ranging from 9.6 × 10^–4^ to 416.9 mW cm^−2^. The suppressed dark current of 2.9 × 10^–11^ A can be attributed to the high crystallization quality and low defect density of the black-phase *γ*-CsPbI_3_ perovskite films. Figure 4c shows the illumination power-dependent photocurrent and responsivity, presenting a linear power dependence. The calculation equation for the responsivity is expressed as *R* = *I−I*_dark_ /*P*, where* I*, *I*_dark_, and *P* are the current, dark current, and illumination power, respectively. The responsivities exhibit a negative dependence with increasing light intensity, which can be attributed to the increased carrier recombination under high illumination power, where the maximum responsivity is 42.1 A W^−1^ corresponding to a light intensity of 9.6 × 10^–4^ mW cm^−2^ at 5 V bias. The linear dynamic range (LDR) is another important parameter to evaluate the performance of the photodetector, which is calculated as LDR = 20 log(*P*_sat_/*P*_low_), *P*_sat_, *P*_low_ representing the highest and lowest illumination power, respectively. The LDR of the device with the DPPOCl additive is 112, which is much larger than the device without the DPPOCl additive with an LDR of 65 (Fig. S11). The long-cycle on–off cycling stability test further demonstrates the excellent cycling stability of the device with the DPPOCl additive (Figs. 4d and S12). The response speed is shown in Figs. 4e and S13, in which the rise (fall) speed is defined as the rise (fall) of the photocurrent from 10% (90%) to 90% (10%) of the maximum current. The high crystallinity and low defect density guarantee a faster response speed with a rise time of 290 µs and a fall time of 340 µs for the device with the DPPOCl additive.

To rigorously evaluate the performance of the photodetector, frequency-dependent noise currents were measured (Fig. 4f). At low-frequency regions, the noise current values are frequency dependent, indicating a 1/*f* dominant noise current. At the high-frequency region, the noise current approaches the shot-noise limit, which is calculated as *I*_shot_ = (2*eI*_dark_)^1/2^, where *e* is the elementary charge. At the same frequency, the noise current of perovskite films without DPPOCl additive is higher than that of perovskite films with DPPOCl additive because of more defects within the film without DPPOCl additive. The normalized response was measured to evaluate the response bandwidth of the device (Fig. 4g). The perovskite films with the DPPOCl additive had a larger 3 dB bandwidth of 1850 Hz than the sample without the DPPOCl additive, which is consistent with the response speed. To evaluate the reproducibility of the devices, we statistically measured the responsivity from ten different devices (Fig. 4h). Furthermore, in a combination of statistical responsivities and noise currents, we systematically compared the detectivity of the devices. The detectivity can be calculated by D* = *R*(*AB*)^1/2^/*i*_noise_, where *A*, *B*, and *i*_noise_ are the operating area of the device, the bandwidth, and the noise current, respectively. The statistical responsivity (detectivity) of perovskite films with the DPPOCl additive is 41.9 ± 2.5 A W^−1^ ((1.3 ± 0.07) × 10^14^ Jones), which is much higher than that of the samples without DPPOCl additive with a statistical responsivity (detectivity) of 0.36 ± 0.21 A W^−1^ ((0.6 ± 0.34) × 10^12^ Jones) at 30 Hz (Fig. S14). Environmental stability is another important parameter for photodetectors. Figure S15 shows the statistical responsivities for ten different devices, perovskite devices without DPPOCl additive show a rapid decrease in responsivity, while perovskite devices with DPPOCl additive maintain more than 60% responsivity under air humidity of 40% for 72 h, which demonstrates better environmental stability due to its high crystallinity, stronger bonding energy, and effective passivation of surface defects. Overall, compared to the perovskite devices without DPPOCl additive and previously reported all-inorganic CsPbI_3_ perovskite film devices, perovskite devices with DPPOCl additive exhibit better optoelectronic performance (Fig. 4i, and Table S1).

### Characterization of Flexible Wearable Devices

**Fig. 5:**
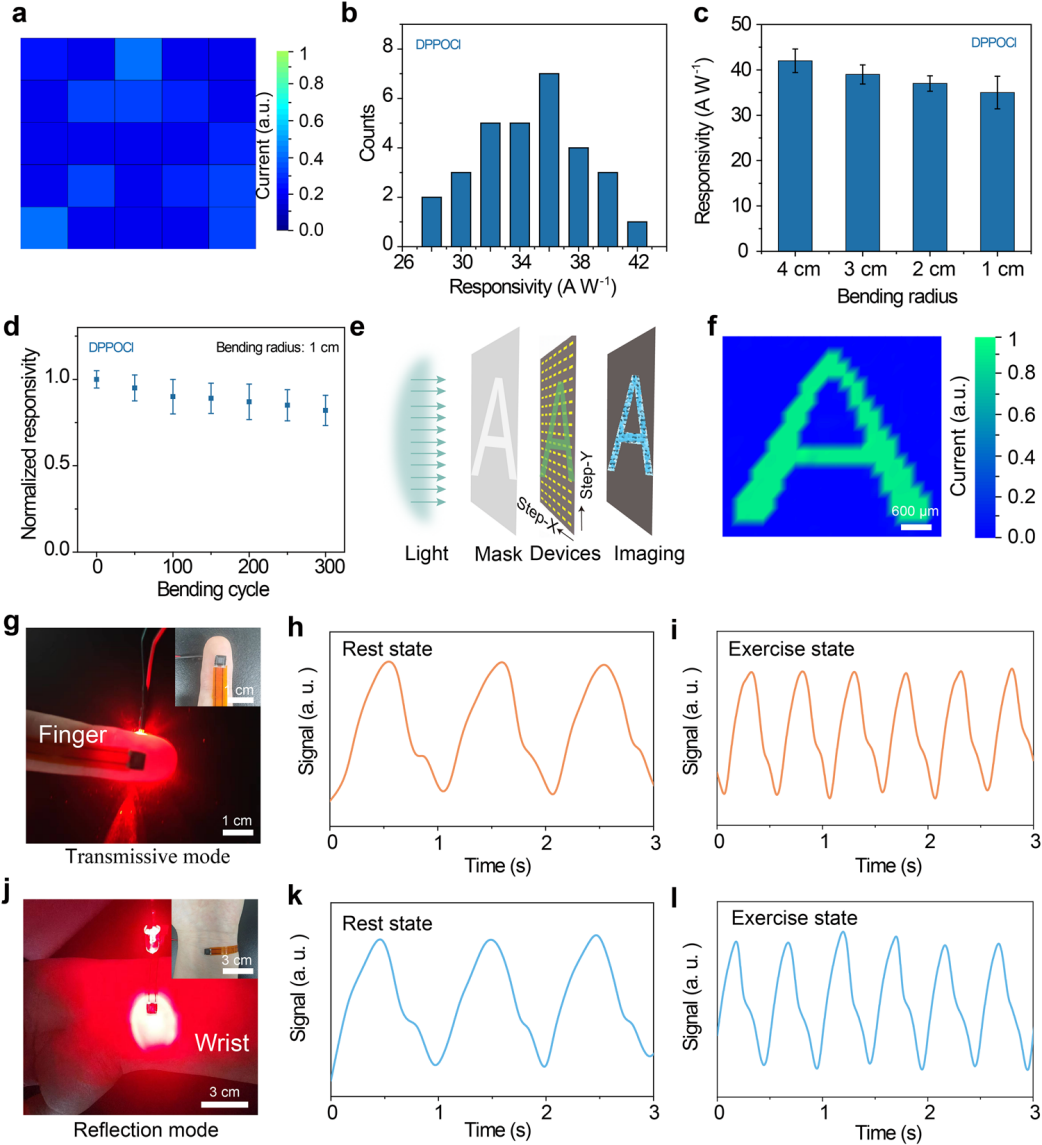
Performance characterization of low-temperature-processed flexible wearable perovskite film devices. **a** Statistical normalized photocurrent imaging map of flexible devices under a fixed light intensity of 416.9 mW cm^−2^, demonstrating uniform crystallinity of perovskite film with DPPOCl additive. **b** Statistical responsivities from thirty different flexible perovskite film devices with DPPOCl additive. Statistical responsivities for flexible perovskite film devices with DPPOCl additive under different **c** bending radii ranging from 1 to 4 cm and **d** bending cycles with a fixed light intensity of 9.6 × 10^–4^ mW cm^−2^ at 5 V bias. **e** Schematic diagram of flexible device imaging for letter A. **f** Imaging result of the letter A demonstrates high fidelity of flexible perovskite film device with DPPOCl additive. PPG sensor in **g** transmissive mode and **j** reflection mode. Heart rate monitoring **h** at rest state and **i** exercise state in transmissive mode. Heart rate monitoring **k** at rest state and **l** exercise state in reflection mode, indicating the high-fidelity pulse signal of the PPG sensor

Given that our method enables the fabrication of high-quality black-phase *γ*-CsPbI_3_ perovskite films at low temperatures, we then investigated the performance of flexible photodetectors. The fabrication of flexible photodetectors is mainly attributed to the realization of low-temperature-processed all-inorganic *γ*-CsPbI_3_ perovskite films. Low-temperature-processed perovskite films possess the following advantages. First, most flexible substrates are not tolerant to high temperatures and are susceptible to deformation at high temperatures. The low-temperature fabrication does not damage the properties of flexible substrates and allows for the efficient integration of flexible devices. Second, compared to high-temperature annealing processes, low-temperature-processed perovskite films can reduce energy consumption and minimize the cost of large-area flexible perovskite devices. With the addition of DPPOCl, we successfully fabricated large-area all-inorganic perovskite flexible films with an area exceeding 25 cm^2^ and can be further scaled up (Fig. S16). Large-area perovskite flexible films also present a smooth surface, indicating their high crystallinity and uniform film deposition. To further demonstrate the uniform crystallinity of the low-temperature-processed flexible perovskite films, we measured the photocurrents from different regions (Fig. 5a). The small difference among different regions demonstrates that the perovskite films with the DPPOCl additive have uniform crystallinity. In contrast, the normalized photocurrents of the perovskite film devices without the DPPOCl additive exhibited significant inhomogeneity, indicating ununiform crystallinity, which is consistent with the SEM result (Fig. S17). To evaluate the performance of flexible perovskite photodetectors with DPPOCl and without DPPOCl additive, we systematically measured the responsivities and detectivity for thirty different devices (Figs. 5b and S18). Compared to the poor performance of flexible perovskite photodetectors without the DPPOCl additive, the calculated responsivities and detectivities for flexible perovskite photodetectors with the DPPOCl additive are 37.7 ± 3.5 A W^−1^ and (1.0 ± 0.10) × 10^14^ Jones, respectively, which are comparable to the rigid devices, indicating that the flexible fabrication will not sacrifice their optoelectronic performance. Furthermore, we studied the mechanical stability of ten different flexible photodetectors under different bending radii and different bending times. The photodetectors exhibited excellent mechanical stability without significant performance degradation after different bending radii and bending cycle experiments under a fixed light intensity of 9.6 × 10^–4^ mW cm^−2^ at 5 V bias (Fig. 5c, d). Compared to the devices with DPPOCl additives, noticeable changes in *I*–*V *curves were demonstrated for the devices without DPPOCl additives before and after the 100 bending cycles test, which can be attributed to the instability of black-phase perovskite without DPPOCl additives, suffering from the damage water and oxygen within the air during the mechanical bending process (Fig. S19). Based on high-performance large-size flexible photodetectors with uniform device performance, a high-fidelity image is eventually achieved (Fig. 5e, f).

To further evaluate the wearable performance of the flexible photodetector, we performed a test of the PPG sensor. Figure 5g, j presents the two modes of PPG sensing testing, transmission mode and reflection mode. The blood pulse signals of the human body at rest state and exercise state were measured to demonstrate the reliability of the PPG device (Fig. 5h, i, k, l). The realization of high-fidelity pulse signals using transmission mode and reflection mode can be attributed to the high signal-to-noise ratio of the flexible photodetectors. The calculated resting and exercising blood pulse frequencies are 63 and 120 Hz, respectively. The above experimental results demonstrate the feasibility of all-inorganic perovskite film devices for PPG sensing, which provides a new research route for the fabrication of all-inorganic flexible wearable devices under low-temperature and low-power consumption conditions.

## Conclusion

In this work, we realized the large-area fabrication of all-inorganic black-phase *γ*-CsPbI_3_ perovskite films using a low-temperature solution processing method for the first time. The chloride ions and DPPOH released by in situ hydrolysis reaction efficiently passivated the defects in the perovskite films, yielding high-performance photodetectors with a responsivity of 42.1 A W^−1^, a detectivity of 1.3 × 10^14^ Jones, and a fast response speed of 290 us. Furthermore, we achieved the fabrication of flexible wearable photodetectors with high mechanical stability and realized high-fidelity imaging and PPG sensors based on the advantage of low-temperature solution processing. This work provides new insight into the fabrication of all-inorganic perovskite thin films based on low-temperature solution processing, which will contribute to the development of large-area flexible perovskite optoelectronic devices, such as tandem solar cells, light-emitting diodes, biosensor, etc.

## Supplementary Information

Below is the link to the electronic supplementary material.

Supplementary file1 (DOCX 3603 kb)
