# Supplementary material for: Low-Temperature Fabrication of Stable Black-Phase CsPbI3 Perovskite Flexible Photodetectors Toward Wearable Health Monitoring
Source: Nanomicro Lett. 2024 Nov 15;17:63. doi: 10.1007/s40820-024-01565-4 (PMC11568160; doi:10.1007/s40820-024-01565-4)
Supplement: Supplementary file 1 — Supplementary file1 (DOCX 3603 kb) [file 40820_2024_1565_MOESM1_ESM.docx]

Supporting Information for

**Low-Temperature Fabrication of Stable Black-Phase CsPbI_3_ Perovskite Flexible Photodetectors towards Wearable Health Monitoring**

Yingjie Zhao^1,^*, Yicheng Sun^1^, Chaoxin Pei^1^, Xing Yin^1^, Xinyi Li^3^, Yi Hao^1^, Mengru Zhang^1^, Meng Yuan^3^, Jinglin Zhou^4^, Yu Chen^4^, and Yanlin Song^2,^*

^1^College of Chemistry and Pingyuan Laboratory, Zhengzhou University, Zhengzhou 450001, P. R. China

^2^Key Laboratory of Green Printing, Institute of Chemistry, Chinese Academy of Sciences, Beijing 100190, P. R. China

^3^Key Laboratory of Bio-inspired Materials and Interfacial Science, Technical Institute of Physics and Chemistry, Chinese Academy of Sciences, Beijing 100190, P. R. China

^4^Institute of High Energy Physics, Chinese Academy of Sciences, Beijing 100049, P. R. China

*Corresponding authors. E-mail: [zhaoyingjie5@zzu.edu.cn](mailto:zhaoyingjie5@zzu.edu.cn) (Yingjie Zhao); [ylsong@iccas.ac.cn](mailto:ylsong@iccas.ac.cn) (Yanlin Song)

**Supplementary Figures**


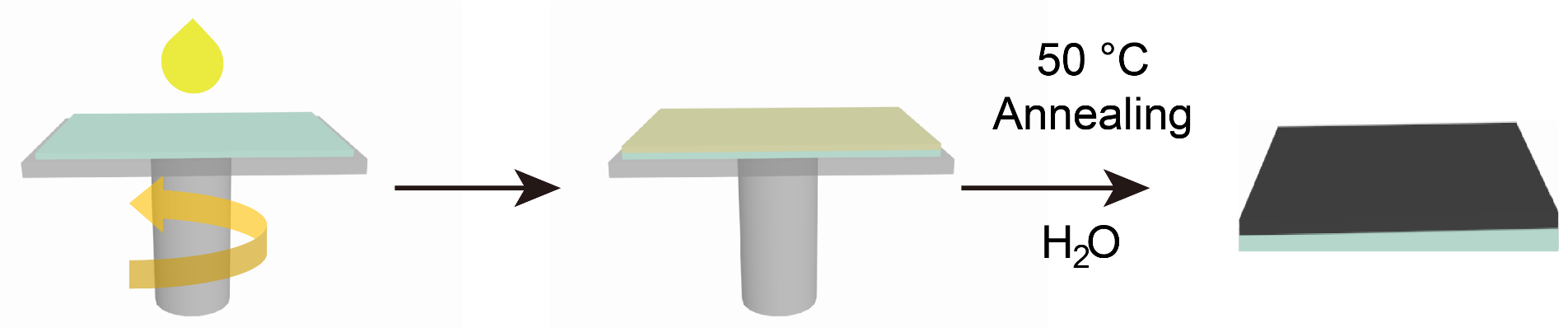


**Fig. S1** Schematic diagram of the preparation process of black-phase CsPbI_3_ perovskite films


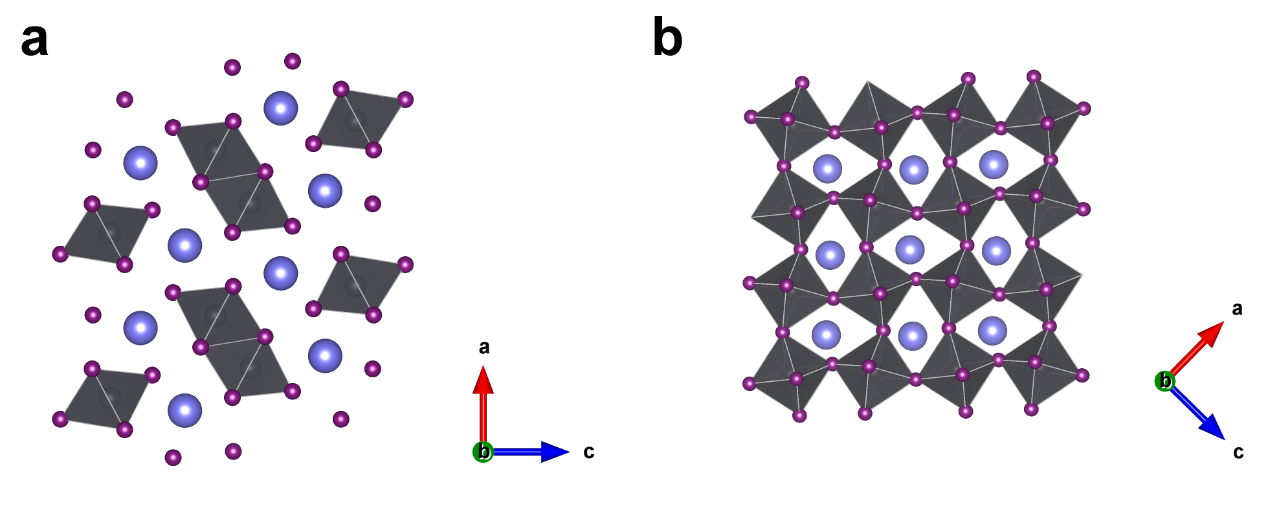


**Fig. S2** Schematic diagram of the crystal of the **a** *δ*-phase and the **b** black-phase perovskite


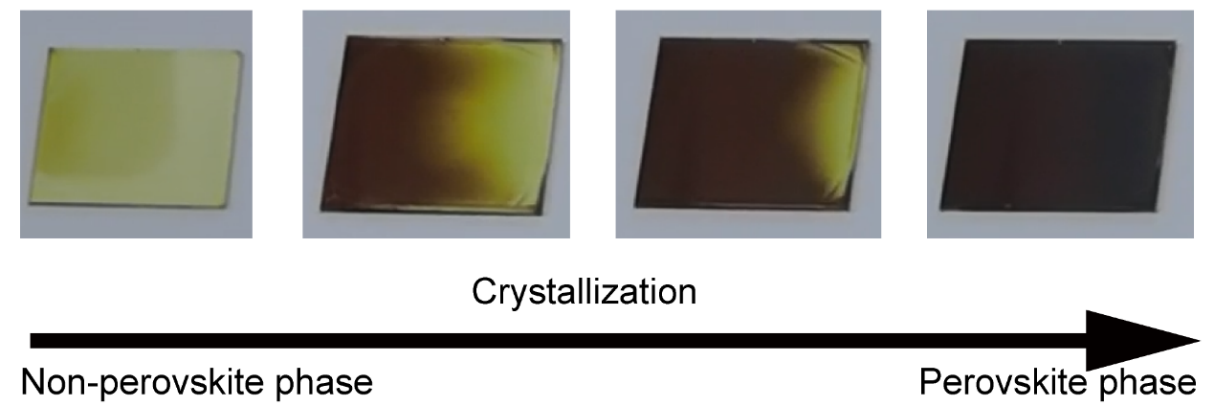


**Fig. S3** Color evolution of perovskite films with DPPOCl additive at different annealing times under 50 ℃


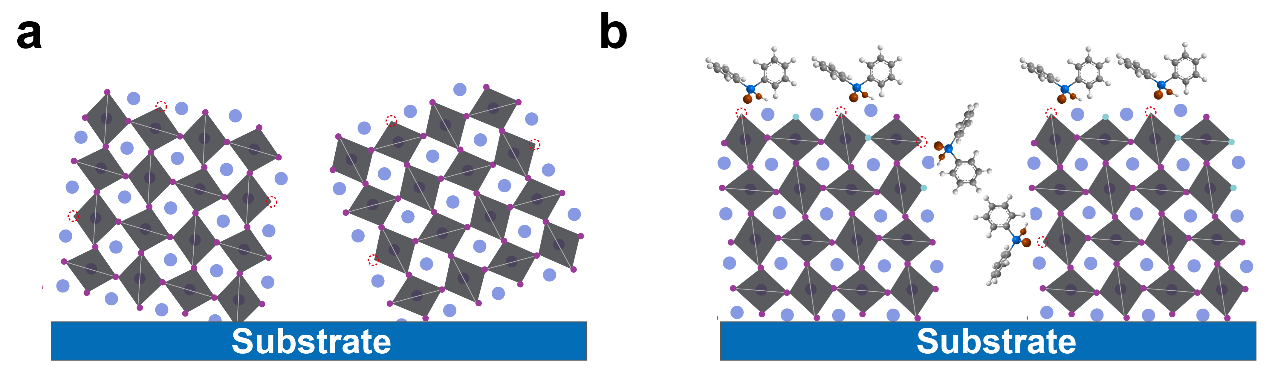


**Fig. S4** Schematic diagram of crystal orientation for perovskite films **a** without the DPPOCl additive and **b** with the DPPOCl additive


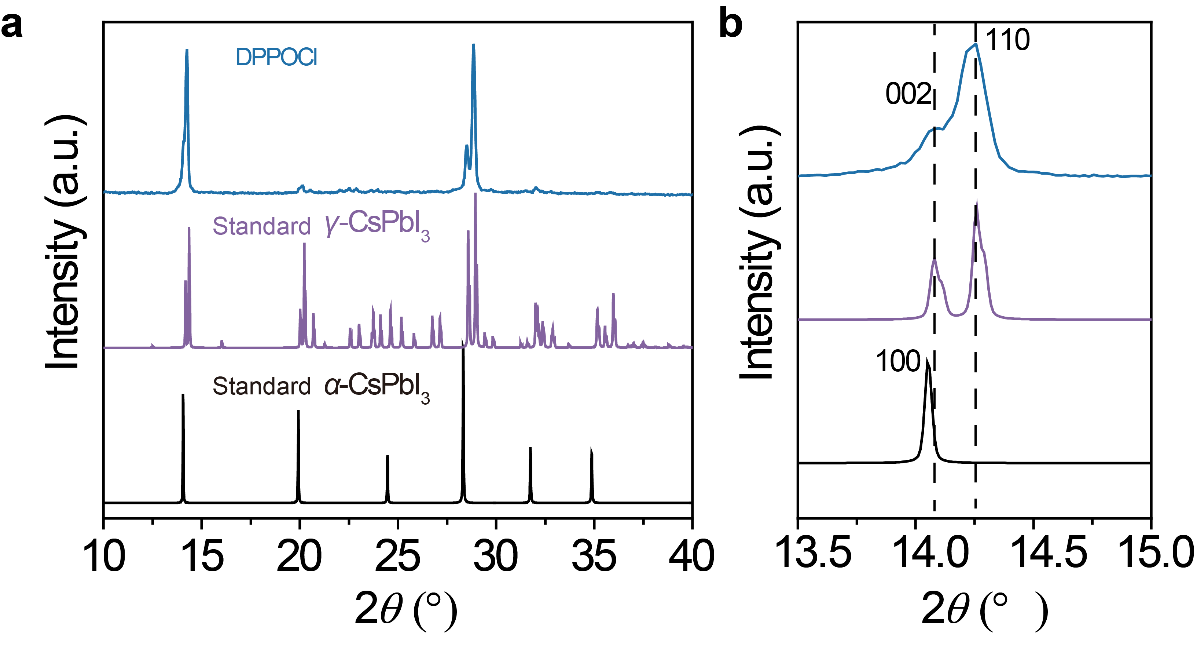


**Fig. S5** XRD patterns of the fabricated perovskite films and the standard diffraction peaks of *α*-CsPbI_3_ phase and*γ*-CsPbI_3_ phase, confirming the formation of the *γ*-CsPbI_3_ phase


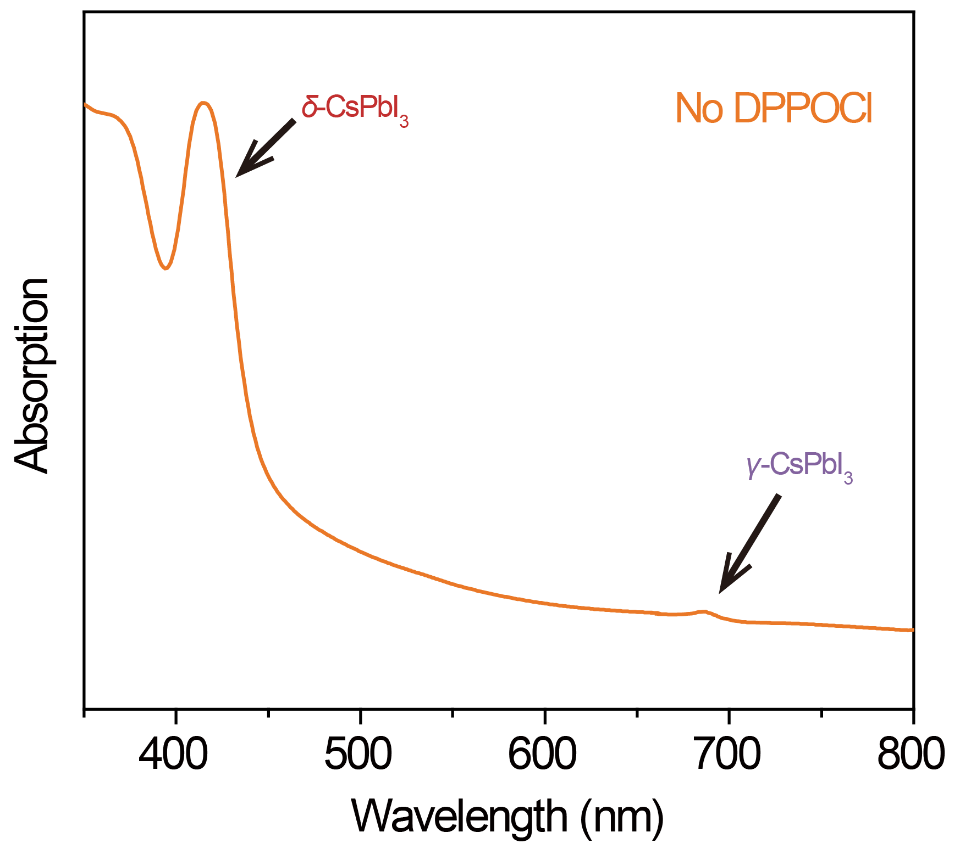


**Fig. S6** The absorption spectra of perovskite film without the DPPOCl additive under an annealing temperature of 50 ℃


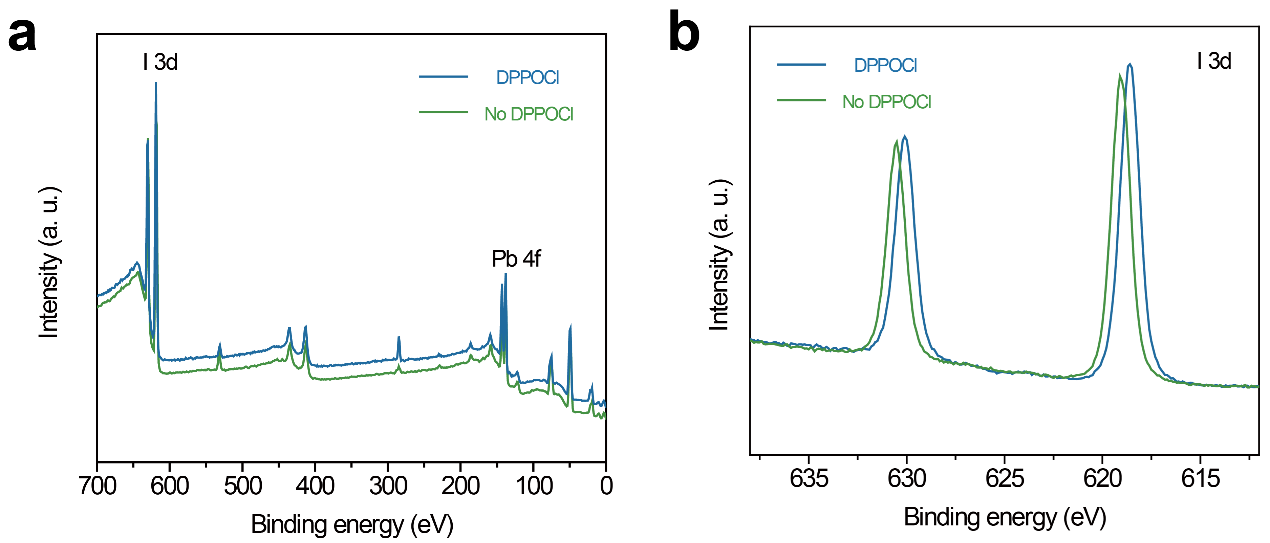


**Fig. S7** XPS spectra of perovskite films with the DPPOCl additive and without the DPPOCl additive


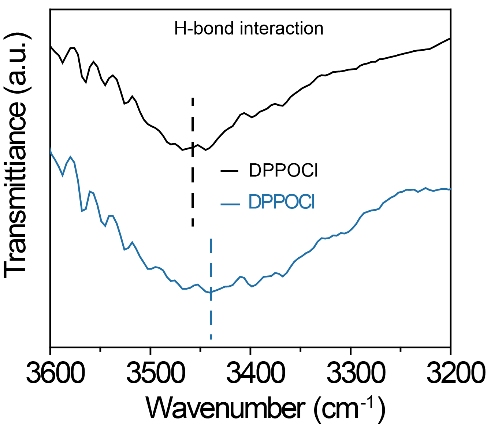


**Fig. S8** The FTIR spectra result of H-bond for perovskite film with DPPOCl additive (blue line) and DPPOCl film (black line)


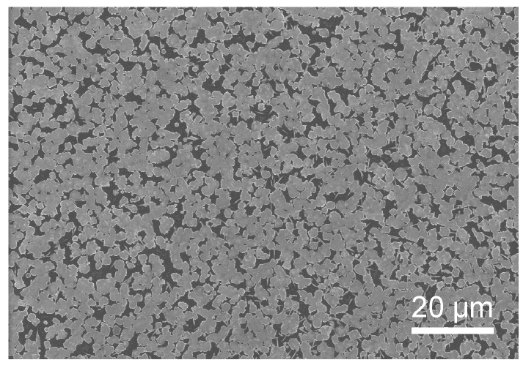


**Fig. S9** The morphology of perovskite film without the DPPOCl additive under an annealing temperature of 50 ℃


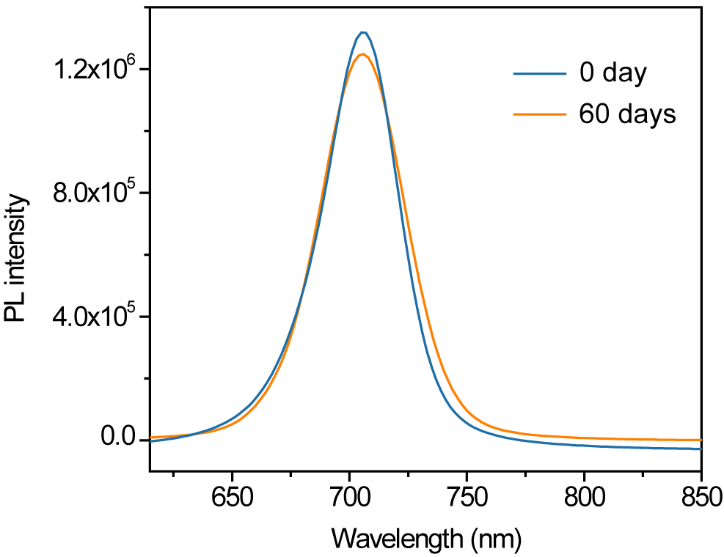


**Fig. S10** The PL stability of perovskite films with the DPPOCl additive at nitrogen atmosphere


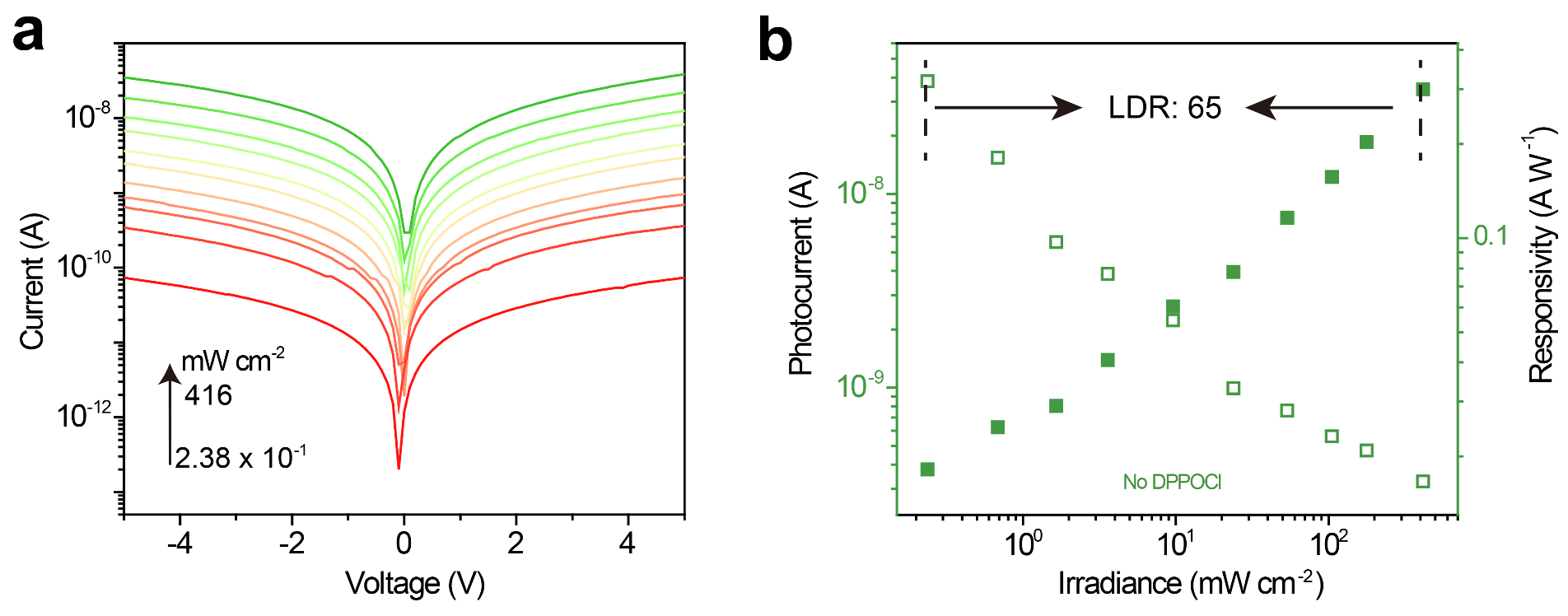


**Fig. S11** **a** Logarithmic *I-V* curve of the photodetector based on perovskite devices without the DPPOCl additive. **b** Photocurrent and responsivities of perovskite devices without the DPPOCl additive under different irradiation powers ranging from 2.38 × 10^-1^ to 416 mW cm^−2^


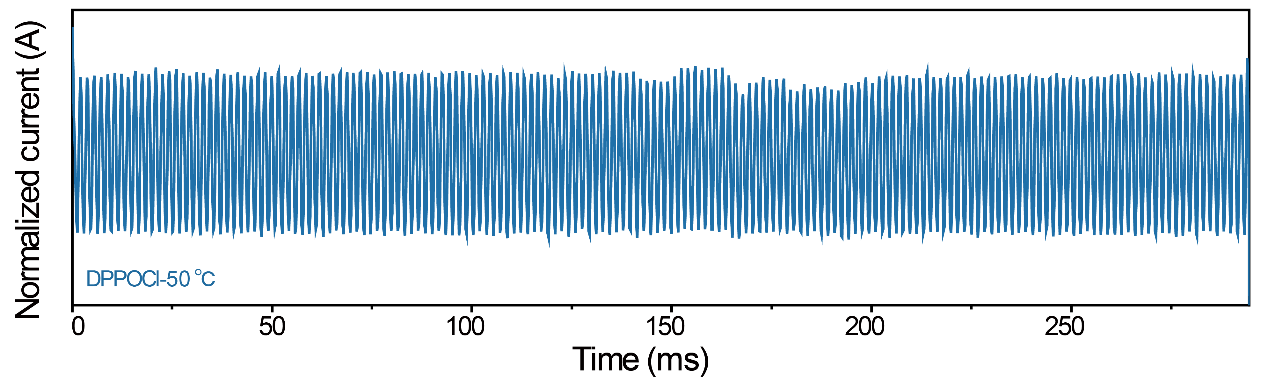
**Fig. S12** On-off stability tests of 176 cycles for perovskite devices with DPPOCl additive under a fixed irradiation power of 416.9 mW cm^−2^ at 5V bias


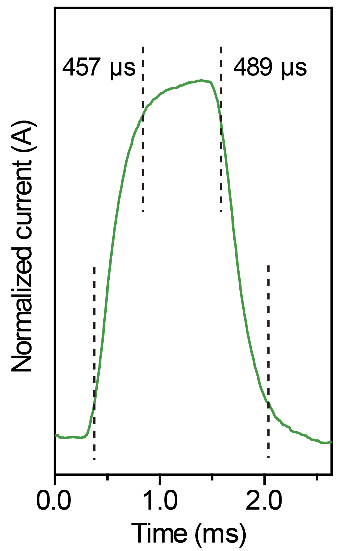


**Fig. S13** Response speed for perovskite devices without the DPPOCl additive under a fixed irradiation power of 416.9 mW cm^−2^ at 5V bias


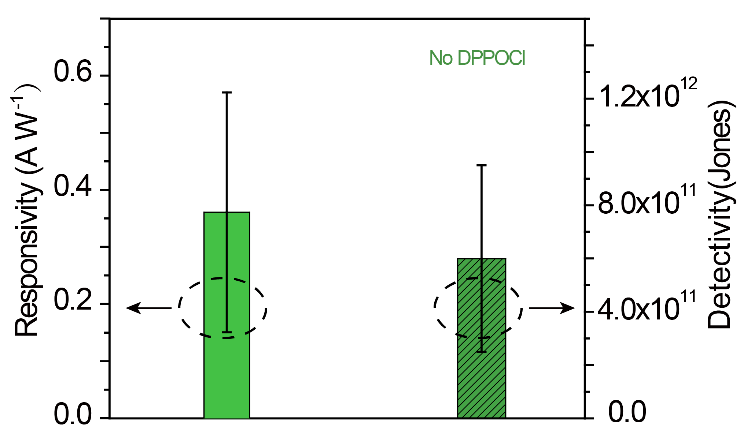


**Fig. S14** Statistical responsivities and detectivities from ten different devices under a fixed light intensity of 2.38 × 10^-1^ mW cm^−2^. Error bars represent standard deviation


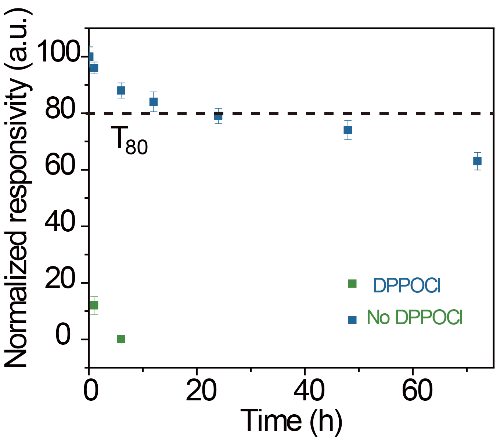


**Fig. S15** Normalized responsivities from ten different devices in the air (humidity: 40%; temperature: 25℃) for different times, indicating that devices with the DPPOCl additive possess superior stability


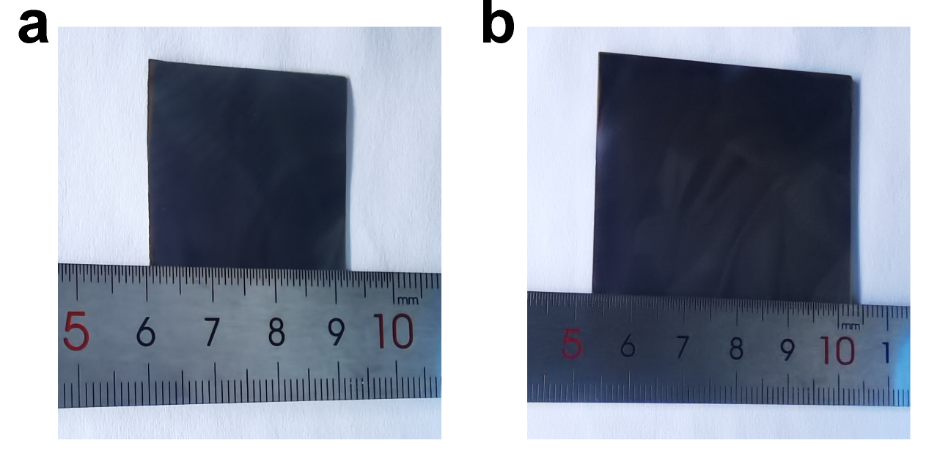


**Fig. S16** Photograph of large-size perovskite films with the DPPOCl additive


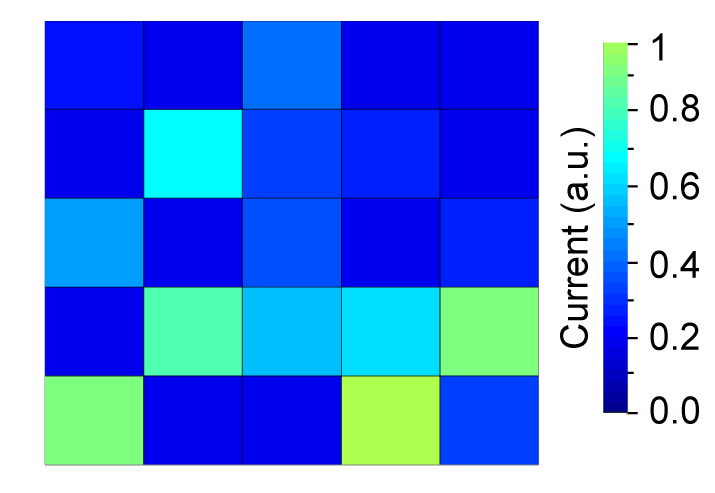


**Fig. S17** Statistical normalized photocurrent imaging map of flexible perovskite film devices without the DPPOCl additive under a fixed light intensity of 416.9 mW cm^−2^


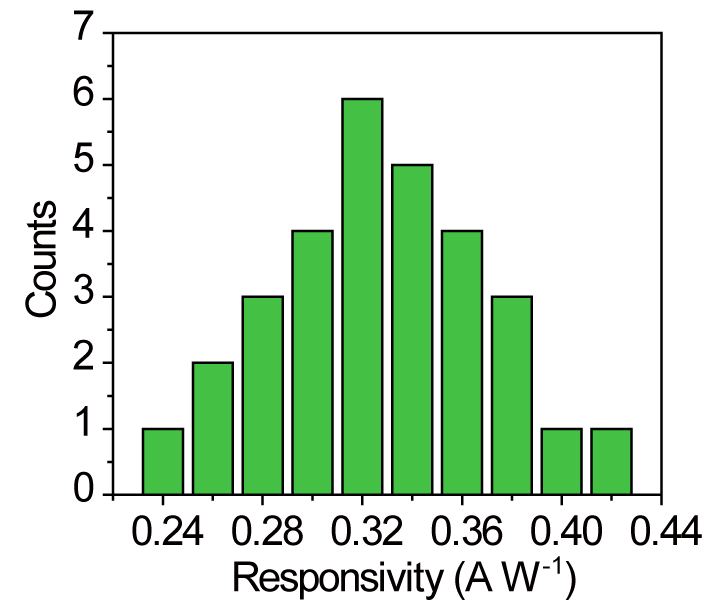


**Fig. S18** Statistical responsivity from thirty different flexible perovskite film devices without the DPPOCl additive


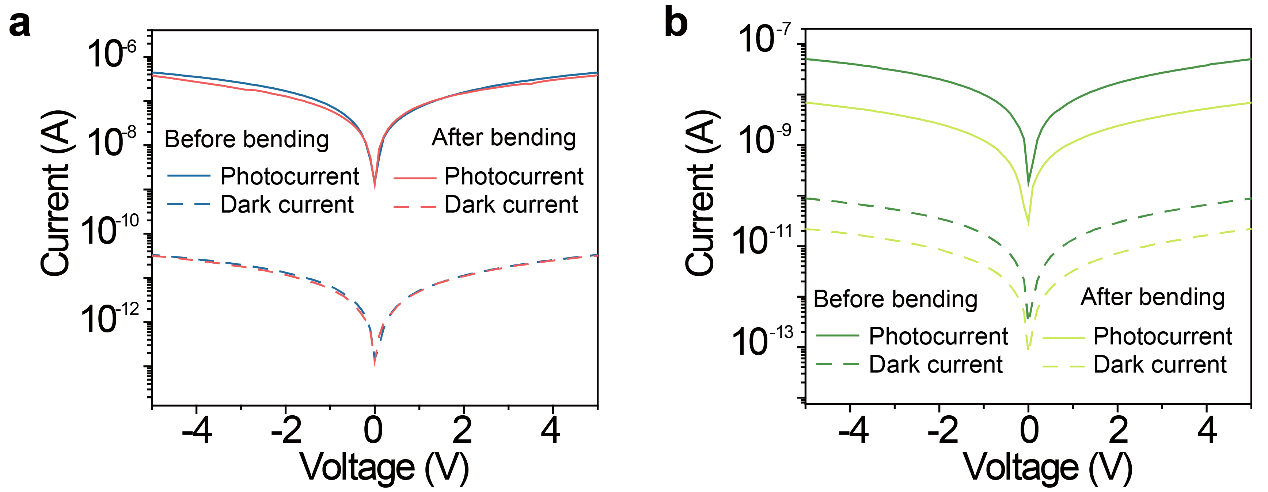


**Fig. S19** Logarithmic *I-V* curve of the photodetector before bending test and after 100 bending cycles test based on perovskite devices. **a** with DPPOCl additive and **b** without DPPOCl additive under a fixed irradiation power of 416.9 mW cm^−2^ and dark conditions

**Supplementary Table**

**Table S1** Device performance parameters comparison between previously reported all-inorganic CsPbI_3_ perovskite film photodetectors and the present perovskite devices

| Device structure | Substrate | Responsivity (A W^-1^) | Detectivity (Jones) | Response  speed  (rise/decay) | Wavelength (nm) | Refs. | |
| --- | --- | --- | --- | --- | --- | --- | --- |
| photoconductor | flexible | 42.1 | 1.3 × 10^14^ | 290.0/340.0 μs | 680 | **This work** |  |
| photodiode | rigid | 0.2 | 2.0 × 10^10^ | 2.0/2.0 μs | 690 | [S1] |  |
| photoconductor | rigid | 3.0 × 10^-4^ | 1.2 × 10^9^ | 2.4/1.1 s | solar light | [S2] | |
| photodiode | rigid | 0.43 | 2.2 × 10^11^ | 0.28/1.1 ms | 550 | [S3] | |
| photodiode | rigid | 0.035 | 1.8 × 10^12^ | / | 640 | [S4] | |
| photodiode | rigid | 8.2 | 1.4 × 10^12^ | 25/30.6 μs | 300-700 | [S5] | |
| photodiode | rigid | 0.16 | 2.7 × 10^12^ | 13.9/12.7μs | 690 | [S6] | |
| photoconductor | flexible | 0.13 | / | 6.0/7.1 ms | 650 | [S7] | |
| photodiode | rigid | 0.1 | 5.0 × 10^13^ | / | 450 | [S8] | |
| photodiode | rigid | 0.4 | 2.4 × 10^12^ | 7 μs/8 μs | 650-680 | [S9] | |
| photoconductor | flexible | 0.75 | 3.5 × 10^10^ | / | 530 | [S10] | |

**Supplementary References**

1. J. A. Steele, T. Braeckevelt, V. Prakasam, G. Degutis, H. Yuan, et al. An embedded interfacial network stabilizes inorganic CsPbI_3_ perovskite thin films. Nat. Commun. **13**(1), 7513 (2022). https://doi.org/10.1038/s41467-022-35255-9
2. K. Maity, U. Pal, H. K. Mishra, P. Maji, P. Sadhukhan, et al. Piezo-phototronic effect in highly stable CsPbI_3_-pvdf composite for self-powered nanogenerator and photodetector. Nano Energy. **92**, 106743 (2022). https://doi.org/10.1016/j.nanoen.2021.106743
3. J. Li, G. Zhang, Z. Zhang, J. Li, Z. Uddin, et al. Defect passivation via additive engineering to improve photodetection performance in CsPbI_2_Br perovskite photodetectors. ACS Appl. Mater. Interfaces **13**(47), 56358-56365 (2021). https://doi.org/10.1021/acsami.1c19323
4. K. M. Sim, A. Swarnkar, A. Nag, D. S. Chung. Phase stabilized *α*‐CsPbI_3_ perovskite nanocrystals for photodiode applications. Laser Photonics Rev. **12**(1), 1700209 (2017). https://doi.org/10.1002/lpor.201700209
5. S. Pal, A. Ghorai, S. Mahato, S. K. Ray. Piezo‐phototronic effect‐induced self‐powered broadband photodetectors using environmentally stable *α*‐CsPbI_3_ perovskite nanocrystals. Adv. Opt. Mater. **11**(16), 2300233 (2023). https://doi.org/10.1002/adom.202300233
6. C. Zhao, P. Liu, W. Cai, W. Xu, M. U. Ali, et al. Polymer‐assisted phase stable *γ*‐CsPbI_3_ perovskite film for self‐powered and ultrafast photodiodes. Adv. Mater. Interfaces. **9**(9), 2102212 (2022). https://doi.org/10.1002/admi.202102212
7. M. Wang, W. Tian, F. Cao, M. Wang, L. Li. Flexible and self‐powered lateral photodetector based on inorganic perovskite CsPbI_3_–CsPbBr_3_ heterojunction nanowire array. Adv. Funct. Mater. **30**(16), 1909771 (2020). https://doi.org/10.1002/adfm.201909771
8. C. Bi, S. V. Kershaw, A. L. Rogach, J. Tian. Improved stability and photodetector performance of CsPbI_3_ perovskite quantum dots by ligand exchange with aminoethanethiol. Adv. Funct. Mater. **29**(29), 1902446 (2019). https://doi.org/10.1002/adfm.201902446
9. M. I. Pintor Monroy, I. Goldberg, K. Elkhouly, E. Georgitzikis, L. Clinckemalie, et al. All-evaporated, all-inorganic CsPbI_3_ perovskite-based devices for broad-band photodetector and solar cell applications. ACS Appl. Electron. Mater. **3**(7), 3023-3033 (2021). https://doi.org/10.1021/acsaelm.1c00252
10. Y. Zhou, J. Luo, Y. Zhao, C. Ge, C. Wang, et al. Flexible linearly polarized photodetectors based on all‐inorganic perovskite CsPbI_3_ nanowires. Adv. Opt. Mater. **6**(22), 1800679 (2018). https://doi.org/10.1002/adom.201800679
